# Preoperative Prognostic Nutritional Index Predicts Long-Term Surgical Outcomes in Patients with Esophageal Squamous Cell Carcinoma

**DOI:** 10.1007/s00268-017-4437-1

**Published:** 2017-12-31

**Authors:** Noriyuki Hirahara, Yoshitsugu Tajima, Yusuke Fujii, Shunsuke Kaji, Tetsu Yamamoto, Ryoji Hyakudomi, Takahito Taniura, Yoshiko Miyazaki, Takashi Kishi, Yasunari Kawabata

**Affiliations:** Department of Digestive and General Surgery, Faculty of Medicine, Shimane University, 89-1 Enya-cho, Izumo, Shimane 693-8501 Japan

## Abstract

**Background:**

The purpose of the present study is to investigate the utility of prognostic nutritional index (PNI) as a simple and readily available marker in esophageal squamous cell carcinoma (ESCC).

**Methods:**

We retrospectively analyzed 169 patients who underwent potentially curative esophagectomy, for histologically verified ESCC. We decided to set the optimal cutoff value for preoperative PNI levels at 49.2, based on the cancer-specific survival (CSS) and the overall survival (OS) by receiver operating characteristic curve analysis.

**Results:**

Multivariate logistic regression analysis identified that TNM pStage III [hazard ratio (HR) 3.261, *p* < 0.0001] and PNI < 49.2 (HR 3.887, *p* < 0.0001) were confirmed as independent poor predictive factors for CSS, and age >70 (HR 2.024, *p* < 0.0042), TNM pStage III (HR 2.510, *p* = 0.0002), and PNI < 49.2 (HR 2.248, *p* = 0.0013) were confirmed as independent poor predictive factors for OS. In non-elderly patients, TNM pStage III (CSS; HR 3.488, *p* < 0.0001, OS; HR 2.615, *p* = 0.0007) and PNI < 49.2 (CSS; HR 3.849, *p* < 0.0001, OS; HR 2.275, *p* = 0.001) were confirmed as independent poor predictive factors for CSS, and OS when multivariate logistic regression analysis was applied. But in elderly patients, univariate analyses demonstrated that the TNM pStage III was the only significant risk factor for CSS (HR 3.701, *p* = 0.0057) and OS (HR 1.974, *p* = 0.0224).

**Conclusions:**

The PNI was a significant and independent predictor of CSS and OS of ESCC patients after curative esophagectomy. The PNI was cost-effective and readily available, and it could act as a marker of survival.

## Introduction

Esophageal cancer is a disease of the elderly, with peak incidence occurring in patients in their 70 s, and the elderly population is rapidly increasing in worldwide. Early detection and treatment confers the greatest chance of long-term survival in patients with esophageal cancer. However, a common problem among esophageal cancer patients (especially the elderly) is low tolerance to the available treatments, despite improvements in surgical techniques and perioperative care with reduced perioperative mortality after esophagectomy. Therefore, there is a continuing interest in prognostic factors to identify patients who are at greater risks of recurrence in order to better tailor individual treatments to those who are more likely to benefit from them. Progression and prognosis of cancer has been shown to be impacted by both the tumor features as well as the patient’s nutritional and immunologic status [1, 2]. Several screening tools are now available for assessing the preoperative nutritional status of patients with cancer, including the subjective global assessment, nutritional risk scoring 2002, body mass index, Glasgow Prognostic Score, and prognostic nutritional index (PNI) [3–6]. Many studies have addressed the association between preoperative nutritional status and postoperative outcomes. However, there is little data to show the impact of nutritional status on long-term outcomes in patients undergoing radical thoracoscopic esophagectomy for esophageal squamous cell carcinoma (ESCC) [7, 8].

The PNI was originally developed to predict the risk of postoperative morbidity and mortality after gastrointestinal surgery [9]. However, the method for calculating PNI is complicated and difficult to use routinely in clinical practice. In contrast, the simplified PNI proposed by Onodera et al. [6] can be easily measured because it is based on only two laboratory parameters, namely the serum albumin level and the peripheral blood lymphocyte count. Albumin is a widely used parameter of nutrition, and its levels have been shown to correlate well with postoperative complications and long-term outcomes in several malignancies [10]. Similarly, reduced lymphocyte count and function also correlate with poor prognosis, by enabling cancer cells to escape immune surveillance [11]. PNI, therefore, is a favorable indicator and is more reflective of the overall status of cancer patients.

In the management of cancer patients, there has been a continuing interest in preoperative prognostic indicators that can allow more accurate patient stratification, precise clinical decision-making, and improvement of short- and long-term outcomes. The TNM classification is widely used as a good standard prognostic indicator of patients with cancer [12]. However, since it includes pathological findings of the tumor, a conclusive classification can only be made postoperatively. In contrast, the PNI is based on the preoperative laboratory data alone and could be a comprehensive indicator of postoperative morbidity, mortality, and prognosis of cancer patients prior to surgery.

Therefore, we have demonstrated the prognostic significance of the simplified PNI in overall and age-stratified ESCC patients with a low *versus* high PNI, and evaluated its potential as a useful surrogate marker for predicting postoperative patient survival.

## Methods

### Patients

We retrospectively reviewed a database containing data from 169 patients who underwent potentially curative esophagectomy with R0 resection, for histologically verified ESCC in our institute, between January 2006 and December 2015. R0 resection was defined as complete tumor removal without the involvement of any microscopic resection margin. Video-assisted or thoracoscopic subtotal esophagectomy with three-field lymph node dissection was performed for all patients, followed by laparoscopic gastric surgery with an elevation of the gastric conduit to the neck via the posterior mediastinal approach or retrosternal approach with an end-to-end anastomosis of the cervical esophagus and gastric conduit. The patients’ clinical characteristics, laboratory data, treatment regimen, and pathological data were obtained from their medical records. None of the patients had clinical signs of infection or other systemic inflammatory conditions preoperatively. In this study, we excluded patients who had received pre- or postoperative adjuvant chemotherapy and/or radiotherapy.

We introduced a perioperative multidisciplinary management team, including surgeon, anesthesiologist, dental hygienist doctor, pharmacist, nutritionist, and rehabilitation technician, and certified expert surgical nurse, which aimed to decrease the incidence rate of postoperative complications. This team mainly managed dental cleaning, medication assistance, physical exercise and rehabilitation, respiratory training, and nutritional support. We provided preoperative enteral nutrition to optimize preoperative condition as possible.

The severity of postoperative complications was evaluated according to the Clavien–Dindo classification, and grade II or higher was recorded as a postoperative complication [13].

We evaluated cancer-specific survival (CSS) and overall survival (OS) as the endpoints of the study. The observation period started from the day of the operation and lasted for up to 5 years, loss to follow-up, withdrawal of consent, or until death. CSS was defined as the interval from the date of operation to the date of cancer-specific death or the last follow-up. Two patients who died of myocardial infarction within 60 days after esophagectomy were excluded from the analysis. We defined ‘elderly’ patients as those aged 70 years or older and ‘non-elderly’ as those aged less than 70 years of age [14].

Permission to perform this retrospective study was obtained from the ethical board of our institution and the study was conducted in accordance with the Declaration of Helsinki.

### Preoperative calculation of the PNI and its cutoff value

The preoperative PNI was calculated using the following formula: 10 × serum albumin (g/dl) + 0.005 × total lymphocyte count (per mm^3^) in peripheral blood [6].

The receiver operating characteristics (ROC) curve of preoperative PNI levels was generated for multiple logistic regression analysis using CSS and OS. The area under the curve (AUC) estimation was used to assess the predictive ability of the PNI.

**Fig. 1:**
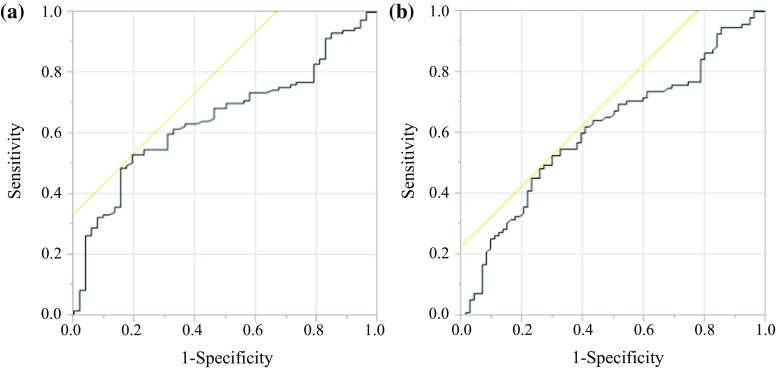
Receiver operating curves for postoperative survival were plotted to verify the optimum cutoff value for PNI. **a** cancer-specific survival, **b** overall survival

We decided to set the optimal cutoff value for preoperative PNI levels at 49.2 in this study, based on the CSS (sensitivity: 52.99%; specificity: 80.77%; AUC of the ROC curve: 0.653) and the OS (sensitivity: 52.63%; specificity: 70.27%; AUC of the ROC curve: 0.6132) at 5 years after surgery (Fig. 1). Based on their PNI values, patients were categorized as having a high PNI (49.2 or greater) or as having a low PNI (less than 49.2).

### TNM pathological Stage (pStage)

The pathological classification of the primary tumor, the degree of lymph node involvement, and the presence of organ metastasis were determined according to the TNM classification system (7th edition of the cancer staging manual of the American Joint Committee on Cancer) [15].

### Statistical analysis

The mean and standard deviation were calculated, and the differences were analyzed using Student’s *t* test. Differences between the various clinicopathological features were analyzed using the Chi-square test. The CSS was analyzed using Kaplan–Meier statistics, and inter-group differences were assessed using the log-rank test.

Univariate analyses were performed to determine variables associated with CSS. Variables with *p* values < 0.05 in the univariate analyses were included in a multivariate logistic regression analysis. The potential prognostic factors assessed were as follows: age (<70 vs. ≥70 years), sex (female vs. male), TNM pStage (I/II vs. III), tumor size (<3 vs. ≥3 cm), operation time (<600 vs. ≥600 min), intraoperative blood loss (<500 vs. ≥500 mL), serum squamous cell carcinoma antigen (SCC) value (<1.5 vs. ≥1.5), and PNI (<49.2 vs. ≥49.2).

All statistical analyses were performed using the statistical software JMP (version 11 for Windows; SAS Institute, Cary, NC), and *p* values <0.05 were considered statistically significant.

## Results

### Relationship between PNI and clinicopathological features

The correlation between the PNI and clinicopathological parameters in the 169 patients enrolled in this study is summarized in Table 1. The preoperative mean value of the PNI in this study was 47.3 ± 6.2, ranging from 26.8 to 65.7. Based on the cutoff value of 49.2, 98 patients (58%) were in the low PNI category and 71 patients (42%) were in the high PNI category. The results of our analysis showed that the PNI value correlated significantly with lymphocyte count (*p* < 0.0001), tumor size (*p* = 0.0327), depth of tumor (*p* < 0.0001), TNM pStage (*p* = 0.0005), SCC antigen level (*p* = 0.0072), albumin level (*p* < 0.0001), and CRP level (*p* = 0.0012).

**Table 1:** Relationships between PNI and clinicopathological features in 169 patients with esophageal cancer

Characteristics	Total patients	PNI		
		<49.2 (*n* = 98)	≥49.2 (*n* = 71)	*p* value
Age (years)		67.1 ± 8.2	65.4 ± 8.0	0.1804
Sex				0.628
Male	150	86	64	
Female	19	12	7	
WBC		5830.0 ± 2404.6	6233.9 ± 1616.6	0.2209
Neutrophil		3773.6 ± 2197.3	3669.2 ± 1264.6	0.7199
Lymphocyte		1396.4 ± 562.7	1931.8 ± 599.2	<0.0001
Platelet		232.8 ± 75.5	225.5 ± 50.2	0.4796
Location of tumor				0.0696
Ce	13	12	1	
Ut	10	6	4	
Mt	73	39	34	
Lt	59	31	28	
Ae	14	10	4	
Tumor size (mm)		5.27 ± 4.98	3.88 ± 2.53	0.0327
Depth of tumor				<0.0001
T1a–1b	72	27	45	
2	13	8	5	
3	67	48	19	
4a–4b	17	15	2	
Lymph node metastasis				0.1573
*N*0	89	49	40	
*N*1	48	28	20	
*N*2	18	9	9	
*N*3	14	12	2	
Pathological stage				0.0005
1a–1b	62	24	38	
2a–2b	39	28	11	
3a–3c	68	46	22	
Operation time (min)		656.1 ± 196.1	646.7 ± 158.6	0.7391
Intraoperative blood loss (ml)		665.5 ± 601.2	580.0 ± 612.3	0.3666
SCC antigen		1.67 ± 2.04	0.97 ± 0.85	0.0072
Albumin		3.64 ± 0.43	4.32 ± 0.30	<0.0001
CRP		0.658 ± 1.098	0.217 ± 0.314	0.0012

### Prognostic factors for postoperative survival in patients with ESCC

Univariate analyses identified advanced TNM pStage [hazard ratio (HR), 4.170; 95% confidence interval (CI), 2.377–7.525; *p* < 0.0001], large tumor size (HR 1.854; 95% CI 1.093–3.678; *p* = 0.0233), prolonged operation time (HR 1.756; 95% CI 1.012–3.035; *p* = 0.0452), and low PNI (HR 4.566; 95% CI 2.375–9.674; *p* < 0.0001) as significant risk factors for shorter CSS. The TNM pStage (HR 3.261; 95% CI 1.808–6.043; *p* < 0.0001) and PNI (HR 3.887; 95% CI 1.999–8.309; *p* < 0.0001) were confirmed as independent prognostic factors for CSS when multivariate logistic regression analysis was applied (Table 2).

**Table 2:** Prognostic factors for cancer-specific survival in patients with esophageal cancer

Variables	Patients (*n* = 169)	Category or characteristics	Univariate			Multivariate		
			HR	95% CI	*p* value	HR	95% CI	*p* value
Gender	19/150	(Female/male)	1.14	0.499–3.290	0.7765			
Age	59/110	(<70/≥70)	1.42	0.790–2.480	0.2344			
TNM pStage	101/68	(1,2/3)	4.17	2.377–7.525	<0.0001	3.261	1.808–6.043	<0.0001
Tumor size	64/105	(<3/≥3)	1.854	1.093–3.678	0.0233	1.076	0.583–2.079	0.8203
Operation time	111/58	(<600/≥600)	1.756	1.012–3.035	0.0452	1.595	0.910–2.786	0.1024
Intraoperative blood loss	87/82	(<500/≥500)	0.973	0.562–1.685	0.9226			
PNI	98/71	(≥49.2/<49.2)	4.566	2.375–9.674	<0.0001	3.887	1.999–8.309	<0.0001
SCC	126/43	(<1.5/≥1.5)	1.169	0.584–2.171	0.6425			

Univariate analyses identified advanced age (HR 1.930; 95% CI 1.205–3.061; *p* = 0.0066), advanced TNM pStage (HR 2.725; 95% CI 1.714–4.349; *p* < 0.0001), large tumor size (HR 1.868; 95% CI 1.149–3.141; *p* = 0.0112), and low PNI (HR 2.612; 95% CI 1.600–4.405; *p* < 0.0001) as significant risk factors for shorter OS. The age (HR 2.024; 95% CI 1.255–3.236; *p* = 0.0042), TNM pStage (HR 2.510; 95% CI 1.555–4.064; *p* = 0.0002), and PNI (HR 2.248; 95% CI 1.362–3.832; *p* = 0.0013) were confirmed as independent prognostic factors for OS in multivariate logistic regression analysis (Table 3).

**Table 3:** Prognostic factors for overall survival in patients with esophageal cancer

Variables	Patients(*n* = 169)	Category or characteristics	Univariate			Multivariate		
			HR	95% CI	*p* value	HR	95% CI	*p* value
Gender	19/150	(Female/male)	1.12	0.550–2.687	0.773			
Age	59/110	(<70/≥70)	1.93	1.205–3.061	0.0066	2.024	1.255–3.236	0.0042
TNM pStage	101/68	(1,2/3)	2.725	1.714–4.349	<0.0001	2.51	1.555–4.065	0.0002
Tumor size	64/105	(<3/≥3)	1.868	1.149–3.141	0.0112	1.219	0.7364–2.083	0.5582
Operation time	111/58	(<600/≥600)	1.481	0.9288–2.344	0.0981			
Intraoperative blood loss	87/82	(<500/≥500)	1.308	0.825–2.098	0.2541			
PNI	98/71	(≥49.2/<49.2)	2.612	1.600–4.405	<0.0001	2.248	1.362–3.832	0.0013
SCC	126/43	(<1.5/≥1.5)	1.589	0.934–2.609	0.0859			

### Prognostic factors for postoperative survival in non-elderly patients with ESCC

Among 110 non-elderly patients, univariate analyses identified advanced TNM pStage (HR 4.646; 95% CI 2.281–10.027; *p* < 0.0001), large tumor size (HR 2.872; 95% CI 1.349–6.825; *p* = 0.0054), and low PNI (HR 11.370; 95% CI 4.040–47.500; *p* < 0.0001) as significant risk factors for shorter CSS. The TNM pStage (HR 3.488; 95% CI 1.948–6.424; *p* < 0.0001) and PNI (HR 3.849; 95% CI 1.987–8.205; *p* < 0.0001) were confirmed as independent prognostic factors for CSS when multivariate logistic regression analysis was applied (Table 4).

**Table 4:** Univariate and multivariate analysis of cancer-specific survival in 110 non-elderly patients with esophageal cancer

Variables	Patients (*n* = 110)	Category or characteristics	Univariate			Multivariate		
			HR	95% CI	*p* value	HR	95% CI	*p* value
Gender	11/99	(Female/male)	0.865	0.307–3.612	0.8142			
TNM pStage	64/46	(1,2/3)	4.646	2.281–10.027	<0.0001	3.488	1.948–6.424	<0.0001
Tumor size	44/66	(<3/≥3)	2.872	1.349–6.825	0.0054	1.115	0.607–2.149	0.7313
Operation time	74/36	(<600/≥600)	1.636	0.307–1.234	0.1664			
Intraoperative blood loss	55/55	(<500/≥500)	1.176	0.591–2.371	0.6437			
PNI	61/49	(≥49.2/<49.2)	11.37	4.040–47.500	<0.0001	3.849	1.987–8.205	<0.0001
SCC	83/27	(<1.5/≥1.5)	1.307	0.516–2.914	0.5471			

Univariate analyses identified advanced TNM pStage (HR 3.772; 95% CI 2.027–7.234; *p* < 0.0001), large tumor size (HR 2.539; 95% CI 1.315-5.298; *p* = 0.0050), and low PNI (HR 3.671; 95% CI 1.864–7.897; *p* = 0.0001) as significant risk factors for shorter OS. The TNM pStage (HR 2.615; 95% CI 1.430–3.768; *p* = 0.0007) and PNI (HR 2.275; 95% CI 1.384–3.863; *p* = 0.001) were confirmed as independent prognostic factors for OS in multivariate logistic regression analysis (Table 5).

**Table 5:** Univariate and multivariate analysis of overall survival in 110 non-elderly patients with esophageal cancer

Variables	Patients (*n* = 110)	Category or characteristics	Univariate			Multivariate		
			HR	95% CI	*p* value	HR	95% CI	*p* value
Gender	11/99	(Female/male)	1.08	0.390–4.478	0.8968			
TNM Stage	64/46	(1,2/3)	3.772	2.027–7.234	<0.0001	2.615	1.430–3.768	0.0007
Tumor size	44/66	(<3/≥3)	2.539	1.315–5.298	0.005	1.281	0.769–2.202	0.3477
Operation time	74/36	(<600/≥600)	1.254	0.661–2.320	0.4803			
Intraoperative blood loss	55/55	(<500/≥500)	1.524	0.822–2.911	0.1821			
PNI	61/49	(≥49.2/<49.2)	3.671	1.864–7.897	0.0001	2.275	1.384–3.863	0.001
SCC	83/27	(<1.5/≥1.5)	1.515	0.697–3.028	0.2787			

### Prognostic factors for postoperative survival in elderly patients with ESCC

In 59 elderly patients, univariate analyses demonstrated that the TNM pStage was the only significant risk factor for shorter CSS and OS (HR 3.701; 95% CI 1.470–9.743; *p* = 0.0057, HR 1.974; 95% CI 0.938–4.038; *p* = 0.0224, respectively) (Table 6).

**Table 6:** Univariate and multivariate analysis of cancer-specific survival and overall survival in 59 elderly patients with esophageal cancer

Variables	Patients (*n* = 59)	Category or characteristics	Cancer-specific survival Univariate			Overall survival Univariate		
			HR	95% CI	*p* value	HR	95% CI	*p* value
Gender	8/51	(Female/male)	1.85	0.526–11.717	0.3755	1.577	0.615–5.349	0.37
TNM pStage	37/22	(1,2/3)	3.701	1.470–9.743	0.0057	1.974	0.938–4.038	0.0224
Tumor size	20/39	(<3/≥3)	0.973	0.390–2.623	0.9542	1.073	0.526–2.313	0.8507
Operation time	37/22	(<600/≥600)	1.909	0.768–4.815	0.1614	1.731	0.859–3.491	0.1235
Intraoperative blood loss	32/27	(<500/≥500)	0.689	0.265–1.714	0.4235	1.023	0.506–2.088	0.9493
PNI	37/22	(≥49.2/<49.2)	1.68	0.655–4.679	0.2838	1.748	0.845–3.784	0.1333
SCC	43/16	(<1.5/≥1.5)	1.218	0.387–3.287	0.715	2.003	0.909–4.214	0.0828

### PNI and postoperative survival in all patients with ESCC

**Fig. 2:**
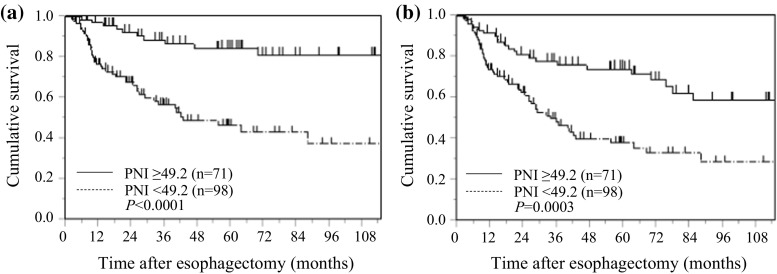
Kaplan–Meier curves of postoperative survival based on PNI in 169 patients with ESCC. **a** cancer-specific survival, **b** overall survival

Kaplan–Meier analysis and the log-rank test demonstrated that patients with a low PNI had a significantly worse prognosis in terms of CSS and OS than those with a high PNI (*p* < 0.0001, and *p* = 0.0003, respectively). The 5-year CSS rates were 46.5 and 84.2%, and the 5-year OS rates were 38.3 and 74.1% for patients with a low PNI and a high PNI, respectively (Fig. 2).

### PNI and postoperative survival in non-elderly patients with ESCC

**Fig. 3:**
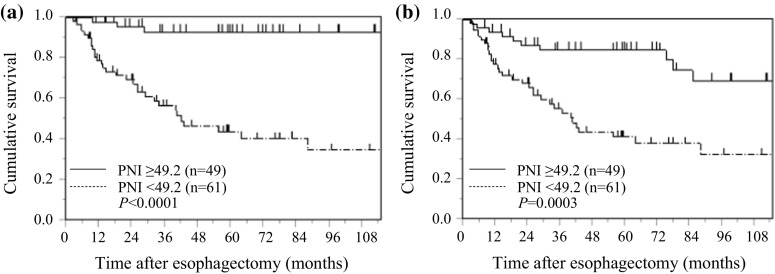
Kaplan–Meier curves of survival based on PNI in 110 non-elderly patients with ESCC. **a** Cancer-specific survival, **b** overall survival

Kaplan–Meier analysis and the log-rank test demonstrated that patients with a low PNI had a significantly worse prognosis in terms of CSS and OS than those with a high PNI (*p* < 0.0001, and *p* = 0.0003, respectively). The 5-year CSS rates were 44.1, and 92.8% and the 5-year OS rates were 41.5 and 84.8% for patients with a low PNI and a high PNI, respectively (Fig. 3).

### PNI and postoperative survival in elderly patients with ESCC

**Fig. 4:**
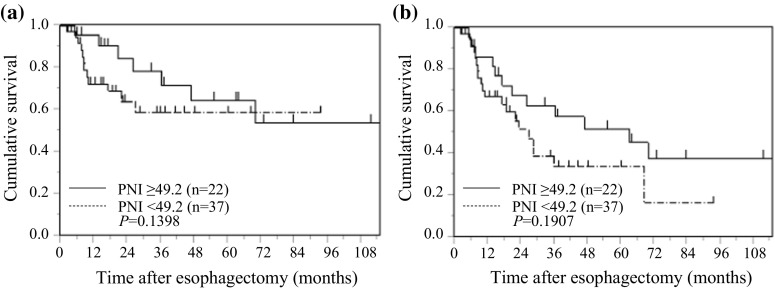
Kaplan–Meier curves of survival based on PNI in 59 elderly patients with ESCC. **a** Cancer-specific survival, **b** overall survival

Kaplan–Meier analysis and the log-rank test showed no significant relationship between PNI and CSS (*p* = 0.1398) or OS (*p* = 0.1907) in elderly patients with ESCC (Fig. 4).

### PNI and postoperative complications

The postoperative complications in the high and low PNI groups are shown in Table 7. No significant differences in incidence rates of postoperative complication were observed between overall and age-stratified patients with a low *versus* high PNI. Similarly, there were no significant differences in postoperative hospital stay (data not shown).

**Table 7:** Comparison between postoperative complications and PNI

	Overall patients	PNI			Non-elderly patients	PNI			Elderly patients	PNI		
		<49.2	≥49.2	*p* value		<49.2	≥49.2	*p* value		<49.2	≥49.2	*p* value
	(*n* = 169) *n* (%)	(*n* = 98) *n* (%)	(*n* = 71) *n* (%)		(*n* = 110) *n* (%)	(*n* = 61) *n* (%)	(*n* = 49) *n* (%)		(*n* = 59) *n* (%)	(*n* = 37) *n* (%)	(*n* = 22) *n* (%)	
Anastomotic leakage	17 (10.1)	9 (9.2)	8 (11.3)	0.657	8 (7.3)	4 (6.6)	4 (8.2)	0.747	9 (15.2)	5 (13.5)	4 (18.2)	0.63
Vocal code paresis	11 (6.5)	6 (6.1)	5 (7.0)	0.811	4 (3.6)	2 (3.3)	2 (4.1)	0.823	7 (11.9)	4 (10.8)	3 (13.6)	0.746
Pulmonary complication	48 (28.4)	30 (30.6)	18 (25.4)	0.454	20 (18.2)	12 (19.7)	8 (16.3)	0.651	28 (47.5)	18 (48.6)	10 (45.5)	0.812
Chylothorax	1 (0.6)	0 (0)	1 (1.4)	0.239	1 (0.9)	1 (1.6)	0 (0)	0.368	0 (0)	0 (0)	0 (0)	NaN
Surgical site infection	10 (5.9)	4 (4.1)	6 (8.5)	0.235	5 (4.5)	2 (3.3)	3 (6.1)	0.477	5 (8.5)	2 (5.4)	3 (13.6)	0.272

*NaN* Not a number

## Discussion

Treatment strategies for ESCC include surgery, radiation, chemotherapy, or a combination thereof. Although a complete surgical resection of the tumor offers a chance for cure, the rate of disease recurrence is very high in ESCC even after an aggressive surgery. Histopathology of surgical specimens is widely used to estimate the prognosis after surgery [12]; however, its predictive value is still limited. The precise evaluation of the risk of postoperative recurrence is important in planning a customized risk-adapted therapeutic strategy for individual patients. In particular, identifying prognostic factors prior to surgery is important to determine the optimal preoperative therapy, and to improve postoperative short- and long-term outcomes [16]. The original PNI consists of tests measuring albumin, transferrin, triceps skin fold, and skin sensitivity reaction to common antigens [17], and it was developed to assess the perioperative complications, including anastomotic leakage, delayed tissue repair, and length of hospital stay in patients undergoing gastrointestinal surgery [11, 18]. The simplified PNI, on the other hand, consists of only two laboratory parameters, namely serum albumin concentration and total lymphocyte count in the peripheral blood [6], both of which are measured routinely in clinical practice. Recently, several studies have reported that albumin is produced by hepatocytes and is regulated by pro-inflammatory cytokines, including interleukin-1 (IL-1), IL-6, and tumor necrosis factor-α (TNF-α) that adversely affect catabolic metabolism [19–21]. These cytokines produced by either the tumor itself or the host are crucial for carcinogenesis, cancer progression, and neo-angiogenesis. In addition, albumin has been shown to help stabilize cell growth and DNA replication, buffer a variety of biochemical changes and maintain sex hormone homeostasis to protect against tumorigenesis [10]. Albumin, therefore, is reflective of the inflammation and immune status of cancer, although it alone is not sufficient to predict the final outcome in cancer patients. Another element of the simplified PNI is the lymphocyte count. Lymphocytes are one of the fundamental components of cell-mediated immunity with inhibitory effects on the proliferation and invasion of tumor cells via cytokine-mediated cytotoxicity [11, 22]. Most patients with ESCC are malnourished, either due to poor dietary intake, protein loss from the primary lesion or due to catabolic metabolism [23]. Impaired nutritional and immunologic status accelerates tumor progression due to a decline in tumor immunity [16, 22]. The PNI is, therefore, a comprehensive indicator of the nutritional and immunologic status in cancer patients [24].

In this study, we evaluated the potential of simplified PNI as a prognostic marker of CSS and OS in ESCC patients who underwent radical thoracoscopic esophagectomy. Patients with a low PNI (less than 49.2) showed a significantly shorter CSS and decreased OS in comparison with patients who had a high PNI (49.2 or greater). This is consistent with our finding that PNI is inversely related to the stage of cancer, the value being lower in patients with a larger, deeper and more aggressive tumor at an advanced TNM pStage [25]. Recent studies have also demonstrated an association between the occurrences of postoperative complications with deteriorated CSS in patients with gastrointestinal cancer [26, 27]. Patients with a low PNI are, therefore, more likely to develop postoperative complications [11, 17, 18], including a systemic inflammatory response, which can also contribute to the decreased CSS and OS. However, this correlation between PNI and CSS/OS was found only in non-elderly patients and not in the elderly patients. This difference could be due to the fact that irrespective of being a cancer patient or not, hypoalbuminemia and leukocytopenia are often associated with aging, leading to malnutrition and immune suppression [28]. Previous studies have reported that cancer patients experiencing postoperative complications generally have a poorer prognosis [29–31]. Because patients with a low preoperative PNI value are at a high risk of postoperative complications, the preoperative PNI value may affect both postoperative short- and long-term outcomes. However, in this study, no significant differences were observed between the PNI value and the rate of postoperative complications. Therefore, further studies are thus needed to address this issue.

The optimal cutoff points for PNI in predicting postoperative survival in patients with malignant tumors is still controversial. Thus, one of the aims of this study was to evaluate the prognostic value of the PNI and propose an optimal cutoff, which can predict CSS and OS with better accuracy, in patients with ESCC. Based on our study of 169 patients who underwent curative esophagectomy, and using a ROC curve, we have arrived at 49.2 as the cutoff value for PNI. The cutoff value for PNI in ESCC patients seems to be much lower than those reported in another cancer, such as renal cell carcinoma, lung cancer, and gliomas [32–34]. The possible reason may be that most ESCC patients have some degree of swallowing difficulty due to obstruction or stricture of the esophagus, which results in the eating disorder, malnutrition, and subsequent poor nutritional status [35].

Several limitations should be considered when interpreting the results of this study. The retrospective nature and a single-institution study, the involvement of a comparatively small sample size, and short follow-up periods were the major limitations of this study. Minor limitations included insufficient evidence of the validity of the cutoff values for the PNI. Regarding the PNI, a fixed cutoff value has not yet been established, and various values have been used in previous reports. Another limitation was that some other parameters involved in systemic inflammation, such as TNF-α and interleukins were not studied, since, these inflammatory parameters are not routinely tested in clinical practice caused by its high cost and inconvenience.

Previous studies have shown that perioperative immunonutrition support improves both nutritional and immunologic status of patients undergoing elective surgeries, thereby reducing postoperative morbidity and mortality [9, 36]. However, it is still unknown whether such nutritional intervention can help improve the surgical outcomes in patients with a low PNI. A larger randomized multicenter prospective study is required to help determine this.
